# Extreme environments as potential drivers of convergent evolution by exaptation: the Atacama Desert Coastal Range case

**DOI:** 10.3389/fmicb.2012.00426

**Published:** 2012-12-19

**Authors:** Armando Azua-Bustos, Carlos González-Silva, Cristián Arenas-Fajardo, Rafael Vicuña

**Affiliations:** ^1^Department of Molecular Genetics and Microbiology, Facultad de Ciencias Biológicas, Pontificia Universidad Católica de ChileSantiago, Chile; ^2^Centro de Investigación del Medio Ambiente, Universidad Arturo PratIquique, Chile

**Keywords:** Atacama Desert, convergent evolution, photrophic eukaryotes

## Abstract

We have recently discovered a variety of unrelated phototrophic microorganisms (two microalgae and one cyanobacteria) in specialized terrestrial habitats at The Coastal Range of the Atacama Desert. Interestingly, morphological and molecular evidence suggest that these three species are all recent colonists that came from aquatic habitats. The first case is Cyanidiales inhabiting coastal caves. Cyanidiales are microalgae that are commonly found in warm acid springs, but have also been recently discovered as cave flora in Italy. The case is *Dunaliella* biofilms colonizing spider webs in coastal caves; *Dunaliella* are microalgae typically found in hypersaline habitats. The third case is *Chroococcidiopsis*, a genus of Cyanobacteria commonly found in deserts around the world that has also been described in warm springs. Thus, we show that the traits found in the closest ancestors of the aforementioned species (which inhabited other unrelated extreme environments) seem to be now useful for the described species in their current subaerial habitats and may likely correspond to cases of exaptations. Altogether, the Coastal Range of the Atacama Desert may be considered as a place where key steps on the colonization of land by phototrophic organisms seem to be being repeated by convergent evolution of extant microalgae and Cyanobacteria.

*The beauty of nature lies in detail; the message, in generality*.

Stephen Jay Gould - *Wonderful Life*.

## Introduction

Convergent evolution of similar phenotypic characteristics in unrelated phylogenetic lineages present in comparable environments has been considered by many as evidence of adaptation. (Conway Morris, 2003). According to Jonathan Losos, “*Convergence in taxa occupying similar selective environments often is the result of natural selection*” (2011). Along with other researchers of the field, we also hold the view that convergent evolution is a portentous example of how natural selection generates the most advantageous solutions to biological problems (one could also say *opportunities*) repetitively presented by the environment. One very appealing group of such environments are those presenting extreme conditions, that is, environments having one or more parameters showing values permanently close to the lower or upper limits known for life. However, when one considers such environments, the adaptation or exaptation question arises. By definition, an adaptation is a trait that evolves due to natural selection in a given environment, whereas an exaptation event provides enhanced fitness in this same environment, but did not initially evolve in response to natural selection in that particular environment (Gould and Vrba, 1982; Losos, 2011). To distinguish between these alternatives, one would need to recreate the original conditions in which a trait evolved, thus testing the role of selection in the evolutionary origin of the trait (Losos, 2011). Interestingly, for the case of convergent exaptations, natural selection may still be involved in favoring the trait in its new environment. As it is not possible to go back 1200 million years ago when aquatic phototrophic species were evolving to colonize the land (the focus of this work), the Atacama Desert Coastal Range arises as an unforeseen option, as will be seen below. In addition, the general approach presented here may offer the unique advantage of providing a potential way to distinguish adaptive from exaptative origins by carefully looking at the species found in all sorts of extreme environments.

## The atacama desert coastal range, a unique interface for the evolution of phototrophic species

### About the atacama coastal range

The Atacama Desert, located in northern Chile, is well known as the driest and oldest desert on Earth. It has experienced aridity for the past 150 million years and hyperaridy for the last 15 million years (Houston and Hartley, 2003; Hartley et al., 2005). The Atacama is characterized by various concurrent harsh conditions, very low air humidity, a nearly complete absence of rains and high-flux solar radiation (Houston and Hartley, 2003; Navarro-González et al., 2003; Hartley et al., 2005). Annual average sunlight irradiation in the Atacama core is 335 W·m-2 with daily maxima over 1000 W·m-2 (McKay et al., 2003). In addition, its soils contain chemically aggressive sulfates, chlorides and perchlorates (Catling et al., 2010). These environmental constraints have caused that extensive regions of the Atacama Desert seem to be devoid of microbial life, with abundances of one or two orders of magnitude below those found in any other arid region on Earth (Navarro-González et al., 2003).

**Figure 1:**
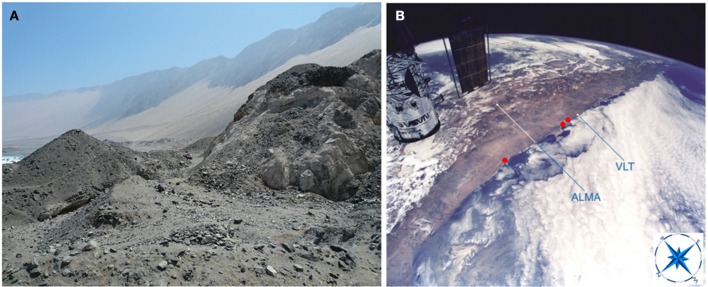
**The Atacama Desert Coastal Range. (A)** The Coastal Range runs along the western margin of the Atacama Desert, with its hills falling abruptly on the Pacific Ocean. At the center left, part of the Pacific Ocean can be seen. For scale, a pickup truck can be barely seen at the center of the image. **(B)** View of the Coastal Range sites detailed in this work, as seen from the NASA Space Shuttle during a servicing mission to the Hubble Space Telescope (partly visible at the upper left). ESO's Very Large Telescope (VLT) at Paranal Observatory and the Atacama Large Millimeter/submillimeter Array (ALMA) are shown as reference points. The three red points from left to right, point to the location of the *Cyanidium* cave, the *Dunaliella* cave, and the quartz field, respectively. Modified from an image by Claude Nicollier (ESO).

The Atacama Desert Coastal Range is a 3100 kilometers long mountain range that runs along the western margin of the Atacama. With maximum altitudes of 3100 meters (Figure 1A), its geological origin is much older than that of the close-by Andes Mountains (Morata et al., 2008). With a mean width of just fifty kilometers, the Coastal Range produces a very effective east rain shadow effect, impeding the entrance of clouds coming from the Pacific Ocean (Cereceda et al., 2002) (Figure 1B). In addition, the Andes Mountains also produce a west rain shadow effect. These combined rain shadows are two of the reasons that explain the extreme aridness of the Atacama, which extends from 15 to 30°S, from sea level up to 3500 m (Houston and Hartley, 2003). This property, along with the location of the Atacama within the dry subtropical climate belt and the presence offshore of a cold, upwelling current dating from at least the early Cenozoic, resulted in conditions promoting climatic stability and the development of the hyperaridity of this desert (Hartley et al., 2005).

As part of our research, we have focused on the photrophic species found at the coastal range. In particular, the ones found in caves and hypolithic habitats, as these may offer a higher and more stable source of moisture available for life in the hyperarid conditions of the Atacama.

### The time setting: 1200 million years ago

By the beginning of the Proterozoic, any coast on Earth most certainly looked like the present Atacama Desert Coastal Range (Nesbitt and Young, 1982; Ross and Chiarenzelli, 1985; Stanley, 1999; Deynoux et al., 2004; Porter, 2006; Kah and Riding, 2007). An arid and barren environment, the coastal range is a lifeless and forbidden place for most water-based life forms (McKay et al., 2003; Cereceda et al., 2008, Azúa-Bustos et al., 2012). The challenges offered by land to aquatic phototrophic species would have seemed insurmountable. Desiccating air conditions, the full spectrum of solar radiation, abrupt temperature fluctuations, nutrient acquisition, and the need for a stronger structural support constituted some of the problems that needed to be tackled if land colonization were to be attempted by the descendants of the stromatolite builders. Interestingly, the success of land phototrophic species has been linked to symbiotic associations, such as those with fungi for example (Simon et al., 1993; Heckman et al., 2001).

Fossils from the Proterozoic document the divergence of all major eukaryotic clades (Knoll et al., 2006). Among these are multicellular filaments of the bangiophyte red algae *Bangiomorpha pubescens*, (Butterfield, 2000). Arouri and colleagues (1999) have also proposed a possible chlorophycean affinity for *Multifronsphaeridium pelorium*, a Neoproterozoic acritarch. Other filamentous microfossils like *Paleovaucheria clavata* (German, 1981) and *Proterocladus* sp. (Butterfield et al., 1994) show several characteristics that indicate a potential affinity with the vaucheriacean algae, which are related to the brown algae (Potter et al., 1997). Scale microfossils of the Tindir Group (northwest Canada) also display characteristics reminiscent of the Chromalveolata, in particular chrysophyte and haptophyte algae (Allison and Hilgert, 1986). The earliest fossils attributed to green algae also date from 1200 million years ago (Knoll, 2003). Thus, the full diversity of aquatic phototrophic species seems to have been present in the ecological setting that then witnessed the colonization of land (Knoll et al., 2006). In fact, shales and sandstones of the mid-proterozoic Belt Supergroup (Montana, USA) that were deposited in shallow water to episodically emergent environments show a variety of sedimentary structures consistent with surfaces colonized by microbial mats (Schiever, 1998).

## Atacama cave microalgae; phylogenetic and ultrastructural evidence for the colonization of land

### A first surprise

**Figure 2:**
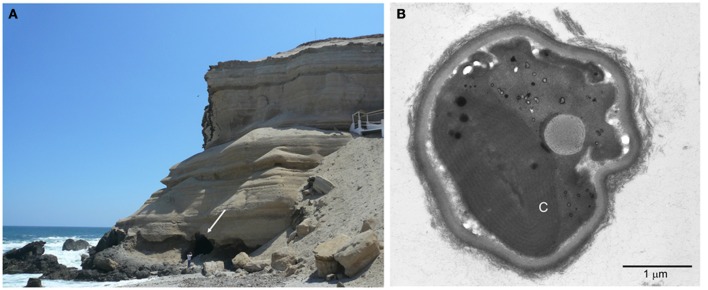
**The *Cyanidium* cave. (A)** Image showing the location of the cave, confronting the Pacific Ocean. The white arrow points to the entrance of the cave. A person stands in the front for scale. **(B)** Transmission electron microscopy micrograph showing the typical ultrastructure of the cave *Cyanidium*. C, chloroplast.

Our original intention was to look for Cyanobacteria that might be found inside caves of the Atacama. The first cave that we described is located at the Coastal Range near the city of Antofagasta (23°38′39 S, 70°24′39 O) (Figure 2A). However, instead of Cyanobacteria, we found a new species of the ancient genus of microalgae *Cyanidium* (Azúa-Bustos et al., 2009). *Cyanidium* species, which are part of the Cyanidiophyceae, are a class of asexual, unicellular red algae usually found in acidic (pH 0.5–3.0) high-temperature (50–55°C) environments such as hot springs (Doemel and Brock, 1970; Ott and Seckbach, 1994). The Cyanidiophyceae were originally classified into three genera, *Cyanidium*, *Cyanidioschyzon*, and *Galdieria* (Ciniglia et al., 2004; Saunders and Hommersand, 2004; Yoon et al., 2006), of which *Cyanidium caldarium* is by far the most studied member. *Cyanidium caldarium* is one the most extremely acidophilic eukaryotes known, with optimum growth conditions of 40–50°C and pH between 1 and 4 (Doemel and Brock, 1971; Beardall and Entwisle, 1984). Consistent with other species of the genus, *Cyanidium* cells found at the Atacama cave are typically rounded, with thick cell walls, a single chloroplast occupying half of the cell interior, a vacuole, and a single mitochondrion (Figure 2B) (Ford, 1984; Albertano et al., 2000). Strikingly, its chloroplast has the structure typical of a cyanobacterium, having phycobilisomes inserted in concentric membranes as opposed to the grana present in more “advanced” algae such as *Chlamydomonas reinhardtii*. This curious internal ultrastructure of *Cyanidium* has been considered as an example of the second endosymbiotic event that gave origin to modern plant cells. In agreement with this assertion, the Cyanidiophyceae have been shown to be one of the most ancient groups of algae, having diverged about 1.3 billion years ago at the base of the Rhodophyta (Seckbach, 1994; Glöckner et al., 2000; Yoon et al., 2002).

By the time of our finding, there were only four known cave *Cyanidium* species, all found in Italy (Ciniglia et al., 2004). Three of these were reported at the Monte Rotaro cave near the island of Ischia, and one at the Sybil cave near Naples. These habitats are non-acidic (pH 7.0–7.2) and non-thermal. These aerophytic epilithic “cave *Cyanidium*” are thought to be the more recent and mesophilic members of the clade, and current phylogenetic analyses support the existence of four distinct lineages: the *Galdieria* spp., the *Cyanidium caldarium*, the *Cyanidioschyzon merolae* plus *Galdieria maxima* lineage, and the novel monophyletic lineage of mesophilic cave *Cyanidium* spp. (Ciniglia et al., 2004). In a broader approach, the group of cave *Cyanidium* supports the hypothesis that life in general evolved from ancient hyperthermophilic species, which then colonized mesophilic environments, and not the other way around (Di Giulio, 2003; Stetter, 2006).

Similar to its cave “cousins”, the Atacama cave *Cyanidium* grows in a mesophilic (15°C) environment with ample water (90% of relative humidity) supplied by the moisture-rich air masses coming from the Pacific Ocean. If an extreme parameter could be defined for the habitat of this species, it would be the light conditions. This species grows in phototrophic biofilms at the bottom of the cave, with 0.06% of the outside incident light. At the bottom of the farthermost zone of the cave, where thin *Cyanidium* biofilms can still be seen, we recorded values of 1 μmol m^−2^ s^−1^ of photosynthetic active radiation (PAR), at the detection limit of the PAR measuring instrument. Thus, this species seems to be very efficient in the use of light. This would explain the observation that these biofilms are seemingly monospecific, containing *Cyanidium* as the sole prototroph.

### A second surprise

**Figure 3:**
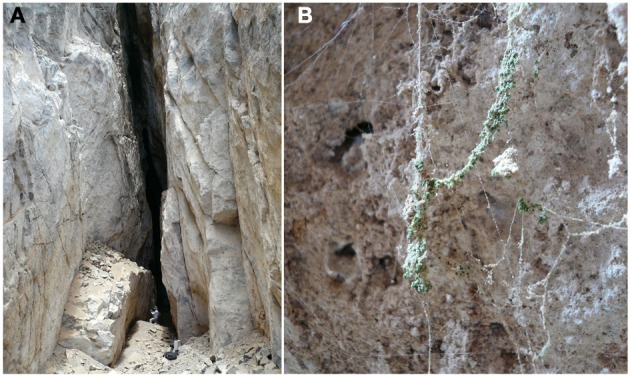
**The *Dunaliella* cave. (A)** Image showing the entrance of the cave, with a person standing in front of the wall where colonized spider web can be found. **(B)** A spider web heavily colonized by *Dunaliella*.

The second coastal cave we analyzed is 255 kilometers further south of Antofagasta (Figure 3A). Also part of the coastal range, the cave is located in the cliffs facing the Pacific Ocean. Similarly to the cave near Antofagasta, moisture rich air masses coming from the Pacific Ocean enter directly into the cave, causing an internal relative humidity of 73%. We analyzed this site as we wanted to find other Cyanidium inhabited caves. Instead, we found a new species of the genus of green algae *Dunaliella* growing at the walls of the cave entrance (Azúa-Bustos et al., 2010). In a wonderful example of biological adaptation, these *Dunaliella* biofilms were found growing almost exclusively upon spider webs attached to the cave walls (Figure 3B). This unusual growth pattern is perfectly understandable if one considers that the cave is located in the driest desert of the world: this *Dunaliella* species was able to use water condensing on the spider web silk threads as way to survive (and we could also say, begin the colonization) of the Atacama Coastal Range. The condensation of water onto threads of spider webs, which are highly hygroscopic (Vehoff et al., 2007), is a common phenomenon, and it can be seen in most household gardens early in the morning. In agreement with this, we recorded the condensation of water in the cave spider webs in the early hours of the morning (Figures 1C,D in Azúa-Bustos et al., 2010).

The surprise stumbled upon in this case is the species itself. Phylogenetic analyses of the 18S rDNA, 16S Rrna, and *psa*B genes (which encode the important D1 subunit part of the photosystem II) revealed these microalgae to be a new species of *Dunaliella*. All 24 previously reported members of this genus are found in extremely saline aquatic environments. Therefore, this new species is the first known subaerial species of the genus. Careful examination of the phylogeny of this species shows that it is highly divergent with respect to other congeneric forms, consistent with the adaptations to its new subaerial habitat (Azúa-Bustos et al., 2010). Interestingly, the species more closely related are found in the Tebenquiche athalassohaline lake (Demergasso et al., 2008), located about 285 km away from the cave, inland in the interior of the Atacama Desert.

**Figure 4:**
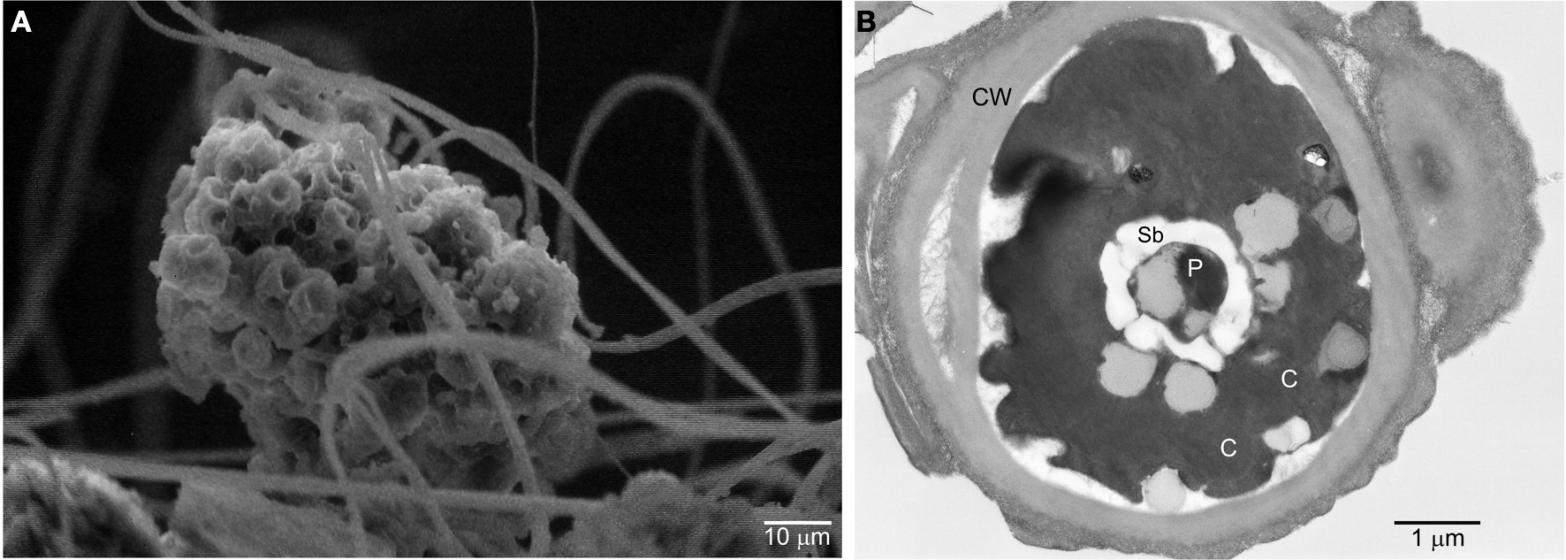
**Scanning and transmission electron micrographs of the cave *Dunaliella*. (A)** SEM micrograph of a group of cells of cave *Dunaliella* attached to the spider web's silk threads. **(B)** TEM micrograph of a cave *Dunaliella* cell. CW, cell wall; C, chlorosplast; P, pyrenoid; Sb, starch bodies.

Typically, aquatic *Dunaliella* species grow as free ranging individuals, as opposed to the cave *Dunaliella*, which grows in colonies. Scanning electron microscopy (SEM) shows one of these colonies firmly attached to the spider web threads (Figure 4A). Interestingly, a stage growth named as the “palmella” stage has been reported for *D. salina* and *D. viridis* subjected to conditions of reduced salinity (Brock, 1975). In the palmella stage, cells lose their flagella, become more rounded and excrete a layer of exopolysaccharides (EPS) in which they repeatedly divide, thus forming colonies (Borowitzka and Silva, 2007). These morphological characteristics are strikingly similar to the ones observed in the colonies of cave *Dunaliella* covering the spider webs. Consistent with this, other phylogenetically close species to our cave *Dunaliella* are two strains of *D. viridis* (SPMO 980625-IE and SPMO202, isolated from the Salt Plains National Wildlife Refuge, Oklahoma, USA) (Assunção et al., 2012).

Some interesting ultrastructural features become evident by transmission electron microscopy (TEM). All remaining aquatic members of this genus have a flexible cell membrane, two flagella and no cell wall. In contrast, our species exhibits a well developed cell wall (Figure 4B) and does not have flagella. This agrees with the hypothesis that unicellular flagellates evolved into non-motile coccoid cells (Leliaert et al., 2012). Intriguingly, in one of the few SEM images where we could see single cells attached to the spider web threads, small stub-like structures reminiscent of flagella were observed (Azúa-Bustos et al., 2010). These stub-like structures are comparable in size to short flagella mutants *of Dunaliella salina* (Vismara et al., 2004) and in the spider web habitat may have a new function as “clinging” devices.

The presence of a cell wall in the cave *Dunaliella* was confirmed by staining with ruthenium red, a dye commonly used to observe acidic pectins and oxidized cellulose, both typical components of cell walls (Sterling, 1970). It has also been previously reported that *Dunaliella salina* strains can produce EPS formed by glucose, galactose, fructose, and xylose in response to increasing salt concentrations (Mishra and Jha, 2009). These sugars have also been shown to be involved in the efficient capture and retention of ambient water by cyanobacteria in extreme subaerial environments (Or et al., 2007; Azúa-Bustos et al., 2011). Certainly, the development of a cell wall would be advantageous for the colonization of land by an aquatic species, as it would of help to better tolerate desiccating conditions, and in addition, provide better structural support.

Another interesting structure seen in TEM images of the Atacama cave *Dunaliella* is a subcellular structure known as pyrenoid (Figure 4B), surrounded by conspicuous starch bodies. Aquatic *Dunaliella* species can only use CO_2_ and bicarbonate as inorganic carbon sources (Hosseini and Shariati, 2009) and since the diffusion of CO_2_ is low in water, aquatic green algae use the pyrenoid to colocalize CO_2_ concentrating mechanisms and the RuBisCo enzymes in order to maximize the fixation of CO_2_ (Kaplan and Reinhold, 1999). The preservation of a pyrenoid in the cells of the cave *Dunaliella* where CO_2_ is not a limiting factor may be a remnant from its former aquatic habitat. Alternatively, the preservation of the pyrenoid might be advantageous for carbon fixation in the new subaerial habitat, as the cell wall may lower CO_2_ diffusion. The distinction of these two alternatives may be crucial for understanding the changes seen in the cave *Dunaliella* as a potential exaptation example.

### A third surprise

**Figure 5:**
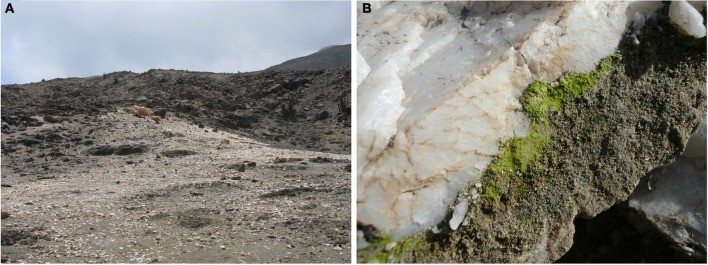
**The quartz field site. (A)** Image showing the quartz outcrop. **(B)** A quartz colonized with a hypolithic biofilm.

Another environment in which our work presently focuses corresponds to the underside of quartz rocks of the Coastal Range near Antofagasta (Figure 5). Here, colonization rates can be up to 80%, with the hypolithic communities thriving with fog as the main regular source of water (Azúa-Bustos et al., 2011). Interestingly, these quartzes have a higher thermal conductivity than the surrounding soils. This results in lower daytime temperatures at the quartz–soil interface microenvironment, favoring water condensation and thus colonization by hypolithic species. In addition, these quartzes can support more than sixty different species, including cyanobacteria, heterotrophic bacteria, archaea, and microalgae (Azúa-Bustos et al., 2012). From one of these biofilms we isolated and cultured a new *Chroococcidiopsis* strain, which we baptized as strain AAB1. *Chroococcidiopsis* is thought to be one of the oldest members of the Cyanobacteria, with its morphology closely resembling Proterozoic microfossils (Friedmann and Ocampo-Friedmann, 1995). Recent phylogenetic analyses also suggest that regional *Chroococcidiopsis* populations share common ancestry dating before the formation of modern continents, thus confirming their antiquity (Bahl et al., 2011).

**Figure 6:**
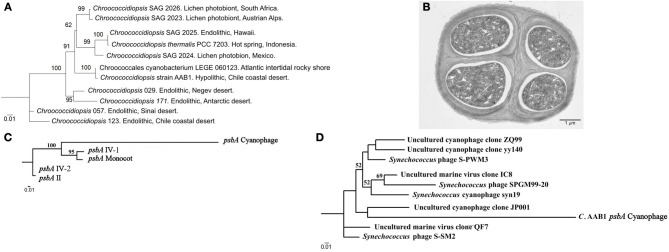
**16S rDNA Maximum Likelihood phylogenetic tree of select *Chroococcidiopsis* 16S rDNA sequences. (A)** 16S rDNA cyanobacterial sequences. Sequences were aligned by Multiple sequence comparison by log-expectation and then analyzed by the PhyML tool of Bosque Phylogenetic Analysis software (Ramírez-Flandes and Ulloa, 2008). Numbers close to the nodes represent 1000 replicate Bootstrap values. Only species shown in Figure 2 of Fewer et al. (2002) were considered for comparison purposes. **(B)** Transmission electron microscopy micrograph showing the typical ultrastructure of a tetrad of *Chroococcidiopsis* strain AAB1. **(C)** Maximum Likelihood phylogenetic trees of *psbA* sequences found in *Chroococcidiopsis* strain AAB1. **(A)** *psbA* sequences found in the genome of *Chroococcidiopsis* strain AAB1. **(D)** A tree showing how a *Chroococcidiopsis* strain AAB1 *psbA* sequence fits in relation with other *psbA* sequences found in marine cyanophages. *psbA* sequences for both trees were aligned by Multiple sequence comparison by log-expectation and then analyzed by the PhyML tool of Bosque Phylogenetic Analysis software. Numbers close to the nodes represent 1000 replicas Bootstrap values.

Phylogenetic analyses with 16 and 23S rDNA gene sequences shows that the strain AAB1 is closer to *Chroococcidiopsis* species found in aquatic environments than to land species (Azúa-Bustos et al., 2012), suggesting its origin from a parental species inhabiting this type of habitat. This assertion is supported by the fact that strain AAB1 is phylogenetically closer to *Chroococcidiopsis thermalis* PCC7203 (isolated from a hot spring in Indonesia) (Geitler, 1933) than to *Chroococcidiopsis* strain 0123 (Figure 6A). This latter strain is an endolithic species also reported from a site at the Coastal Range, which is about 35 kilometers south from where we isolated strain AAB1. Intriguingly, the closest relative of our strain, cyanobacterium LEGE 06123 isolated from intertidal pools in Portugal, (another aquatic-subaerial interface habitat) was suggested to be a species of *Chroogloeocystis*, a proposed new genus (Ramos et al., 2010). In agreement with our phylogenetic analyses, cyanobacterium LEGE 06123 also forms a clade with aquatic strains. Moreover, it also aggregates to form colonies held together by EPS.

A second evidence for the aquatic origin of strain AAB1 is a particular lateral gene transfer event detected in the preliminary analysis of the draft genome of this strain. For this, a short introduction is required; photosystem II is the first complex in the light-dependent reactions, and it is located in the thylakoid membrane of plants, algae, and Cyanobacteria. In the Cyanobacteria, the photosystem II is composed of at least 20 subunits as well as other accessory, light-harvesting proteins. A key subunit of the photosystem II is the D1 protein, encoded by the *psbA* family of genes. The core of the photosystem II complex is composed of the D1 and D2 proteins, which are involved in ligating most of the redox-active components of photosystem II including the Mn_4_Ca cluster, the site of water oxidation in oxygenic photosynthesis (Mulo et al., 2009). In the Cyanobacteria, a small gene family codes for various, functionally distinct D1 isoforms, which can vary from one to four depending on the species (Mulo et al., 2012). In strain AAB1 there are four isoforms, consistent with these reports. In addition, we found a fifth D1 isoform, which is highly divergent from its counterparts (Figure 6C). Strikingly, the *psbA* sequence of this fifth isoform closely resembles sequences found in cyanophages that infect *Synechococcus* species, which are marine Cyanobacteria (Suttle, 2002; Bailey et al., 2004; Huang et al., 2011) (Figure 6D). The closest species of the clade where the AAB1 sequence is positioned (JP001 in Figure 6D) is a phage collected in the Pacific Northeast near Vancouver.

The source of origin of cyanobacterial sequences encoded by cyanophages may be understood if one considers that cyanophages evolved more than 3 billion years ago, about the same time when the Cyanobacteria diverged from other prokaryotes (Suttle, 2002). It is likely that both hosts and phages co-evolved sharing similar selection pressures (Bailey et al., 2004). Thus, *psbA* genes could have been incorporated by error by cyanophages during their replication cycle and then transferred to other Cyanobacteria, constituting a prime example of lateral gene transfer (Sullivan et al., 2005). It remains to be established whether phage *psbA* genes have a function in their new hosts, although some authors have suggested that marine cyanophages may protect their marine hosts against photoinhibition, as a way to ensure a constant energy supply during phage replication (Bailey et al., 2004). If so, one could envision that the maintenance of the cyanophage-related *psbA* sequence in strain AAB1 could be advantageous for the proper adaptation to a hypolithic habitat where the availability of light under the quartz is about 3% of the incident light. In this scenario, the cyanophage-related *psbA* sequence may be expressed under high light conditions in order to avoid photoinhibition, a situation which could be expected in dispersal/colonization events.

An additional circumstance where a functional maintenance of the cyanophage-related *psbA* sequence by strain AAB1 could be advantageous is during desiccation. In phylogenetically related desiccation tolerant cyanobacterial species like *Nostoc commune*, deactivation of photosynthesis can be triggered by the loss of small amounts of water (Hirai et al., 2004). This deactivation arises as photoinhibition quickly results from an imbalance of energy reception and the lack of the water as an electron donor (Fukuda et al., 2008). Thus, in species such as strain AAB1, which were able to colonize the oldest and driest desert of the world, alternative mechanisms like unconventional D1 proteins usage of cyanophage origin could surpass this limitation, allowing photosynthesis under otherwise desiccating conditions.

## The atacama coastal range: a likely scenario for convergent evolution by exaptation?

The collection of observations hereby described was not obtained in order to test a hypothesis. Instead, they emerged as unexpected findings in our research program which is focused on understanding how microbial species of the hyperarid Atacama Desert get access to water.

All phylogenetic analyses show that the closest ancestors of the two unrelated microalgae and one cyanobacterium mentioned here were aquatic species. On the other hand, both morphological and physiological data reveal that these species have developed structures needed to survive in a subaerial environment. This evidence begs questions such as why are these species “jumping out of the water” and why have they chosen to do so in the driest coast on Earth? One would expect that if they were to depart from an aquatic habitat, they would do it in a milder environment, like a beach in the tropics. The answers to these questions probably lie in the careful analysis of the habitats of the closest ancestors of these “rebel” species.

The first consideration is that selection does not actually operate on the inherited traits themselves, but on their functional consequences (Arnold, 1983). Related to this assertion, a common factor for the ancestors of the three studied species is that they evolved in extreme environments characterized by stressful conditions like high salinity, high temperatures or low pH. Thus, these species already had a set of tolerance responses that could alternatively be used in other seemingly unrelated extreme environments; the same mechanisms that allowed ionic stress tolerance caused by salt in *Dunaliella* or *Chroococcidiopsis*, also permitted osmotic stress tolerance caused by desiccation, as both are responses to low water activity. In the same vein, the formation of the palmella stage in some *Dunaliella* species in response to decreasing salt concentrations could be expected to be part of the strategies required for the colonization of land.

Another important factor to be considered is the potentially lesser competition to be found by these species at the Coastal Range due to hyperaridity. The dramatic lack of water causes that the number of free niches available for life in the Atacama is for sure higher compared to a tropical beach, only of course, if the “water problem” can be dealt with. The cave *Dunaliella* fits here in a spectacular way, with the *Cyanidium* and the *Chroococcidiopsis* strains being able to survive in very specific habitats where water availability is assured in an otherwise harsh environment.

As mentioned before, it has been proposed that co-operative interactions with fungi may have helped early plants to adapt to the stresses of the terrestrial realm (Heckman et al., 2001). Interestingly, in the process of isolating the gene sequences required for the identification of the cave *Dunaliella*, we often found the 18S rRNA gene sequence of a species of *Hortaea*, a known halophilic fungi. This finding also fits with our theory. As proposed by Professor Nina Gunde-Cimerman, of the University of Ljubljana, if there is a close association between *Hortaea* and *Dunaliella*, this could be an evidence for the evolution of lichens (pers. comm). It is well known that lichens are one of the first organisms to colonize barren environments at the first stages of a primary ecological succession.

Thus, instead of referring to adaptations of these phototrophic species to a subaerial environment, one (as proposed by Gould) should refer to exaptations. Consistent with this view, the extant species described here still show evidence of the adaptations to their previous habitats, namely, the maintenance of the pyrenoid by *Dunaliella*, the presence of phage-like contained *psbA* sequences from marine origins in *Chroococcidiopsis*, the adaptation to low light levels in *Cyanidium* (which could be expected in aquatic environments), etc.

A particular trait may not be favored by natural selection, even if it confers increased functional capabilities. To provide a fitness advantage, the enhanced capabilities must actually lead to an increase in survival or reproductive success. For the examples shown here, this seems to be the case. One may also consider the time range that took for the first phototrophic species to colonize land. The evolution of the first land plants from land phototrophic species took about 750 million years. In turn, the Atacama has imposed its arid conditions on life for the past 150 million years. Thus, put in a very simplistic way, the collection of adaptations shown by the described *Dunaliella*, *Cyanidium*, and *Chroococcidiopsis* species at the Coastal Range of the Atacama are at the “one fifth milestone” (750/150 my) of the road taken by the ancestors of modern plants. As these three species seem to have crossed the threshold where they are able to live and reproduce on land, they may now be in the evolutionary transit to better adapt to this habitat. If these species are retracing the same path taken by plants, one could expect the next important stage to be the division of tasks in specialized groups of cells, in other words, the origins of multicellularity. Intriguingly, the molecular evidence suggests that *Chroococcidiopsis* is the closest living relative of filamentous heterocyst-differentiating cyanobacteria (Fewer et al., 2002), with the extant species having reverted to unicellularity. Nonetheless, an important stage in the *Chroococcidiopsis* cycle is the tetrad stage (Azúa-Bustos et al., 2012) (Figure 6B). One could then test the hypothesis that there is a functional reason for the organization of cells in tetrads. Could this phenomenon represent a potential and initial division of labor? Consistent with the importance of the close arrangement of seemingly similar cells and the origin of multicellularity is a report in which the genome of the multicellular *Volvox carteri* was compared with the genome of its unicellular relative *Chlamydomonas reinhardtii* (Prochnik et al., 2010). This study revealed that increases in complexity seem to be more associated with modifications of lineage-specific proteins than to large-scale development of protein-coding capacity.

A potential test for the hypothesis of the exaptation cases proposed here is to investigate whether the observed traits actually represent increased functional capabilities. Is this the case, for example, of the phage related D1 protein under photoinhibitory conditions for *Chroococcidiopsis* strain AAB1? In turn, one could test whether these species can still reproduce under the conditions where their ancestors lived.

Another prediction that could be tested is that there should be other extant microorganisms living in this region whose closest ancestors are also species that evolved in extreme aquatic environments. A thorough sampling of the Coastal Range would help to respond to this query. If so, one may also expect similar cases in other extreme environments in which exaptation plays a crucial role, making these extreme environments effective drivers of convergent evolution.

### Conflict of interest statement

The authors declare that the research was conducted in the absence of any commercial or financial relationships that could be construed as a potential conflict of interest.

## References

[B1] Albertano P. Ciniglia C. Pinto G. Pollio A. (2000). The taxonomic position of *Cyanidium*, *Cyanidioschyzon* and *Galdieria*: an update. Hydrobiologia 433, 137–143

[B2] Allison C. W. Hilgert J. W. (1986). Scale microfossils from the Early Cambrian of Northwest Canada. J. Paleontol. 60, 973–1015

[B3] Arnold S. J. (1983). Morphology, performance and fitness. Am. Zool. 23, 347–361 10.1111/j.1558-5646.2008.00343.x 18266988

[B4] Arouri K. Greenwood P. F. Walter M. R. (1999). A possible chlorophycean affinity of some Neoproterozoic acritarchs. Org. Geochem. 30, 1323–1337

[B5] Assunção P. Jaén-Molina R. Caujapé-Castells J. de la Jara A. Carmona L. Freijanes K. (2012). Molecular taxonomy of Dunaliella (Chlorophyceae), with a special focus on *D. salina*: ITS2 sequences revisited with an extensive geographical sampling. Aquat. Biosyst. 8:2 10.1186/2046-9063-8-2 22520929 PMC3310333

[B6] Azúa-Bustos A. González-Silva C. Mancilla R. A. Salas L. Gómez-Silva B. McKay C. P. (2011). Hypolithic cyanobacteria supported mainly by fog in the coastal range of the Atacama Desert. Microb. Ecol. 61, 568–581 21188376 10.1007/s00248-010-9784-5

[B7] Azúa-Bustos A. González-Silva C. Mancilla R. A. Salas L. Palma R. E. Wynne J. J. (2009). Ancient photosynthetic eukaryote biofilms in an Atacama Desert coastal cave. Microb. Ecol. 58, 485–496 10.1007/s00248-009-9500-5 19259626

[B8] Azúa-Bustos A. González-Silva C. Salas L. Palma R. E. Vicuña R. (2010). A novel subaerial Dunaliella species growing on cave spiderwebs in the Atacama Desert. Extremophiles 14, 443–452 10.1007/s00792-010-0322-7 20623153

[B9] Azúa-Bustos A. Urrejola C. Vicuña R. (2012). Life at the dry edge: microorganisms of the Atacama Desert. FEBS Lett. 586, 2939–2945 10.1016/j.febslet.2012.07.025 22819826

[B10] Bahl J. Lau M. C. Smith G. J. Vijaykrishna D. Cary S. C. Lacap D. C. (2011). Ancient origins determine global biogeography of hot and cold desert cyanobacteria. Nat. Commun. 2:163 10.1038/ncomms1167 21266963 PMC3105302

[B11] Bailey S. Clokie M. R. Millard A. Mann N. H. (2004). Cyanophage infection and photoinhibition in marine cyanobacteria. Res. Microbiol. 155, 720–725 10.1016/j.resmic.2004.06.002 15501648

[B12] Beardall J. Entwisle L. (1984). Internal pH of the obligate acidophile *Cyanidium caldarium* Geitler (Rhodophyta?). Phycologia 23, 397–399

[B13] Borowitzka M. A. Silva C. J. (2007). The taxonomy of the genus Dunaliella (Chlorophyta, Dunaliellales) with emphasis on the marine and halophilic species. J. Appl. Phycol. 19, 567–590

[B14] Brock T. D. (1975). Salinity and the ecology of *Dunaliella* from Great Salt Lake. J. Gen. Microbiol. 89, 285–292

[B16] Butterfield N. J. (2000). *Bangiomorpha pubescens* n. gen., n. sp.: implications for the evolution of sex, multicellularity, and the Mesoproterozoic/Neoproterozoic radiation of eukaryotes. Paleobiology 26, 386–404

[B17] Butterfield N. J. Knoll A. H. Swett K. (1994). Paleobiology of the Neoproterozoic Svanberg fjellet Formation, Spitsbergen. Fossils Strata 34, 1–84

[B18] Catling D. C. Claire M. W. Zahnle K. J. Quinn R. C. Clark B. C. Hecht M. H. (2010). Atmospheric origins of perchlorate on Mars and in the Atacama. J. Geophys. Res. 115, E00–E11

[B19] Cereceda P. Larrain H. Osses P. Farías M. Egaña I. (2008). The climate of the coast and fog zone in the Tarapacá Region, Atacama Desert, Chile. Atmos. Res. 87, 301–311

[B20] Cereceda P. Osses P. Larraín H. Farias M. Schemenauer R. S. (2002). Advective, orographic and radiation fog in the Tarapacá Region, Chile. Atmos. Res. 64, 261–271

[B21] Ciniglia C. Yoon H. S. Pollio A. Pinto G. Bhattacharya D. (2004). Hidden biodiversity of the extremophilic Cyanidiales red algae. Mol. Ecol. 13, 1827–1838 10.1111/j.1365-294X.2004.02180.x 15189206

[B22] Conway Morris S. (2003). Life's Solution: Inevitable Humans in a Lonely Universe. Cambridge, UK: Cambridge University Press

[B23] Demergasso C. Escudero L. Casamayor E. O. Chong G. Balagué V. Pedrós-Alió C. (2008). Novelty and spatio-temporal heterogeneity in the bacterial diversity of hypersaline Lake Tebenquiche (Salar de Atacama). Extremophiles 12, 491–504 10.1007/s00792-008-0153-y 18347752

[B24] Deynoux M. Miller J. M. G. Domack E. W. (2004). Earth's Glacial Record. Cambridge: Cambridge University Press

[B25] Di Giulio M. (2003). The universal ancestor was a thermophile or a hyperthermophile: tests and further evidence. J. Theor. Biol. 221, 425–436 10.1006/jtbi.2003.3197 12642117

[B26] Doemel W. N. Brock T. D. (1970). The upper temperature limit of *Cyanidium caldarium*. Arch. Mikrobiol. 72, 326–332 5474131 10.1007/BF00409031

[B27] Doemel W. N. Brock T. D. (1971). The physiological ecology of *Cyanidium caldarium*. J. Gen. Microbial. 67, 17–32

[B28] Fewer D. Friedl T. Büdel B. (2002). *Chroococcidiopsis* and heterocyst-differentiating cyanobacteria are each other's closest living relatives. Mol. Phylogenet. Evol. 23, 82–90 10.1006/mpev.2001.1075 12182405

[B29] Ford T. (1984). A comparative ultrastructural study of *Cyanidium caldarium* and the unicellular red algae Rhodosorus marinus. Ann. Bot. 53, 285–294

[B30] Friedmann E. I. Ocampo-Friedmann R. (1995). A primitive cyanobacterium as pioneer microorganism for terraforming Mars. Adv. Space Res. 15, 243–246 11539232 10.1016/s0273-1177(99)80091-x

[B32] Fukuda S. Y. Yamakawa R. Hirai M. Kashino Y. Koike H. Satoh K. (2008). Mechanisms to avoid photoinhibition in a desiccation-tolerant cyanobacterium, *Nostoc commune*. Plant Cell Physiol. 49, 488–492 10.1093/pcp/pcn018 18252733

[B33] Geitler L. (1933). Diagnosen neuer Blaualgen von den Sunda-Inseln. Arch. Hydrobiol. Suppl. 8, 622.

[B34] German T. (1981). Filamentous microorganisms in the Lakhanda Formation on the Maya River. Paleontol. Zh. 1981, 100–107

[B35] Glöckner G. Rosenthal A. Valentin K. (2000). The structure and gene repertoire of an ancient red algal plastid genome. J. Mol. Evol. 51, 382–390 11040290 10.1007/s002390010101

[B36] Gould S. J. Vrba E. S. (1982). Exaptation – a missing term in the science of form. Paleobiology 8, 4–15

[B37] Hartley A. J. Chong G. Houston J. Mather A. (2005). 150 million years of climatic stability: evidence from the Atacama Desert, Northern Chile. J. Geol. Soc. Lond. 162, 421–424

[B38] Heckman D. S. Geiser D. M. Eidell B. R. Stauffer R. L. Kardos N. L. Hedges S. B. (2001). Molecular evidence for the early colonization of land by fungi and plants. Science 293, 1129–1133 10.1126/science.1061457 11498589

[B39] Hirai M. Yamakawa R. Nishio J. Yamaji T. Kashino Y. Koike H. (2004). Deactivation of photosynthetic activities is triggered by loss of a small amount of water in a desiccation-tolerant cyanobacterium, *Nostoc commune*. Plant Cell Physiol. 45, 872–878 10.1093/pcp/pch094 15295070

[B40] Hosseini T. A. Shariati M. (2009). *Dunaliella* biotechnology: methods and applications. J. Appl. Microbiol. 107, 14–35 10.1111/j.1365-2672.2009.04153.x 19245408

[B41] Houston J. Hartley A. (2003). The central Andean west-slope rainshadow and its potential contribution to the origin of hyper-aridity in the Atacama Desert. Int. J. Climatol. 23, 1453–1464

[B42] Huang S. Wang K. Jiao N. Chen F. (2011). Genome sequences of siphoviruses infecting marine Synechococcus unveil a diverse cyanophage group and extensive phage-host genetic exchanges. Environ. Microbiol. 14, 540–558 10.1111/j.1462-2920.2011.02667.x 22188618

[B42a] Kah L. C. Riding R. (2007). Mesoproterozoic carbon dioxide levels inferred from calcified cyanobacteria. Geology 35, 799–802

[B43] Kaplan A. Reinhold L. (1999). CO2 concentrating mechanisms in photosynthetic microorganisms. Annu. Rev. Plant Physiol. Plant Mol. Biol. 50, 539–570 10.1146/annurev.arplant.50.1.539 15012219

[B44] Knoll A. H. (2003). Life on a Young Planet: The First Three Billion Years of Evolution on Earth. Princeton, NJ: Princeton University Press

[B45] Knoll A. H. Javaux E. J. Hewitt D. Cohen P. A. (2006). Eukaryotic organisms in Proterozoic oceans. Philos. Trans. R. Soc. Lond. B Biol. Sci. 361, 1023–1038 10.1098/rstb.2006.1843 16754612 PMC1578724

[B46] Leliaert F. Smith D. R. Moreau H. Herron M. D. Verbruggen H. Delwiche C. F. (2012). Phylogeny and molecular evolution of the green algae. Crit. Rev. Plant Sci. 31, 1–46

[B47] Losos J. B. (2011). Convergence, adaptation and constraint. Evolution 65, 1827–1840 10.1111/j.1558-5646.2011.01289.x 21729041

[B49] McKay C. P. Friedmann E. I. Gómez-Silva B. Cáceres-Villanueva L. Andersen D. T. Landheim R. (2003). Temperature and moisture conditions for life in the extreme arid region of the Atacama Desert: four years of observations including the El Niño of 1997–1998. Astrobiology 3, 393–406 10.1089/153110703769016460 14577886

[B50] Mishra A. Jha B. (2009). Isolation and characterization of extracellular polymeric substances from micro-algae *Dunaliella salina* under salt stress. Bioresour. Technol. 100, 3382–3386 10.1016/j.biortech.2009.02.006 19272770

[B51] Morata D. Féraud G. Aguirre L. Arancibia G. Belmar M. Morales S. (2008). Geochronology of the Lower Cretaceous volcanism from the Coastal Range (29°20'-30°S), Chile. Rev. Geol. Chile 35, 123–145

[B52] Mulo P. Sakurai I. Aro E. M. (2012). Strategies for psbA gene expression in cyanobacteria, green algae and higher plants: from transcription to PSII repair. Biochim. Biophys. Acta 1817, 247–257 10.1016/j.bbabio.2011.04.011 21565160

[B53] Mulo P. Sicora C. Aro E. M. (2009). Cyanobacterial psbA gene family: optimization of oxygenic photosynthesis. Cell. Mol. Life Sci. 66, 3697–3710 10.1007/s00018-009-0103-6 19644734 PMC2776144

[B54] Navarro-González R. Rainey F. A. Molina P. Bagaley D. R. Hollen B. J. de la Rosa J. (2003). Mars-like soils in the Atacama Desert, Chile, and the dry limit of microbial life. Science 302, 1018–1021 10.1126/science.1089143 14605363

[B55] Nesbitt H. W. Young G. M. (1982). Early Proterozoic climates and plate motions inferred from major element chemistry of lutites. Nature 299, 715–717

[B56] Or D. Phutane S. Dechesne A. (2007). Extracellular polymeric substances affecting pore-scale hydrologic conditions for bacterial activity in unsaturated soils. Vadose Zone J. 6, 298–305

[B57] Ott F. D. Seckbach J. (1994). New classification for the genus Cyanidium Geitler 1933, in Evolutionary Pathways and Enigmatic Algae: Cyanidium caldarium (Rhodophyta) and Related Cells, ed Seckbach J. (London: Kluwer Academic), 145–152

[B59] Porter S. M. (2006). The proterozoic fossil record of heterotrophic eukaryotes, in Neoproterozoic Geolobiology and Paleobiology, 27, eds Xiao S. Kaufman A. J. (Dordrecht, The Netherlands: Springer), 1–21

[B60] Potter D. Saunders G. W. Andersen R. A. (1997). Phylogenetic relationships of the Raphidophyceae and Xanthophyceae as inferred from nucleotide sequences of the 18S ribosomal RNA gene. Am. J. Bot. 84, 966–972 21708651

[B61] Prochnik S. E. Umen J. Nedelcu A. M. Hallmann A. Miller S. M. Nishii I. (2010). Genomic analysis of organismal complexity in the multicellular green alga *Volvox carteri*. Science 329, 223–226 10.1126/science.1188800 20616280 PMC2993248

[B62] Ramírez-Flandes S. Ulloa O. (2008). Bosque: integrated phylogenetic analysis software. Bioinformatics 24, 2539–2541 10.1093/bioinformatics/btn466 18762483

[B63] Ramos V. Seabra R. Brito A. Santos A. Santos C. L. Lopo M. (2010). Characterization of an intertidal cyanobacterium that constitutes a separate clade together with thermophilic strains. Eur. J. Phycol. 45, 394–403

[B64] Ross G. M. Chiarenzelli J. R. (1985). Paleoclimatic significance of widespread proterozoic silcretes in the bear and churchill provinces of the northwestern canadian shield. J. Sediment. Petrol. 55, 196–204

[B65] Saunders G. W. Hommersand M. (2004). Assessing red algal supraordinal diversity and taxonomy in the context of contemporary systematic data. Am. J. Bot. 91, 1494–1507 10.3732/ajb.91.10.1494 21652305

[B66] Schiever J. (1998). Possible indicators of microbial mat deposits in shales and sandstones: examples from the Mid-Proterozoic Belt Supergroup, Montana, U.S.A. Sediment. Geol. 120, 105–124

[B67] Seckbach J. (1994). The first eukaryotic cells – acid hot-spring algae. J. Biol. Phys. 20, 335–345

[B68] Simon L. Bousquet J. Levesque R. C. Lalonde M. (1993). Origin and diversification of endomycorrhizal fungi and coincidence with vascular land plants. Nature 363, 67–69

[B69] Stanley S. M. (1999). Earth System History. New York, NY: W.H. Freeman and Company

[B70] Sterling C. (1970). Crystal-structure of ruthenium red and stereochemistry of its pectic stain. Am. J. Bot. 57, 172–175

[B71] Stetter K. O. (2006). Hyperthermophiles in the history of life. Philos. Trans. R. Soc. Lond. B Biol. Sci. 361, 1837–1842 10.1098/rstb.2006.1907 17008222 PMC1664684

[B72] Sullivan M. B. Coleman M. L. Weigele P. Rohwer F. Chisholm S. W. (2005). Three Prochlorococcus cyanophage genomes: signature features and ecological interpretations. PLoS Biol. 3:e144. 10.1371/journal.pbio.0030144 15828858 PMC1079782

[B73] Suttle C. A. (2002). Cyanophages and their role in the ecology of cyanobacteria, in The Ecology of Cyanobacteria: Their Diversity in Time and Space, eds Whitton B. A. Potts M. (Boston, MA: Kluwer Academic Publishers), 563–589

[B74] Vehoff T. Glisovi A. Schollmeyer H. Zippelius A. Salditt T. (2007). Mechanical properties of spider dragline silk: humidity, hysteresis, and relaxation. Biophys. J. 93, 4425–4432 10.1529/biophysj.106.099309 17766337 PMC2098708

[B75] Vismara R. Verni F. Barsanti L. Evangelista V. Gualtieri P. (2004). A short flagella mutant of *Dunaliella sallina* (Chlorophyta, Cholorophyceae). Micron 35, 337–344 10.1016/j.micron.2004.01.001 15006360

[B76] Yoon H. S. Hackett J. D. Pinto G. Bhattacharya D. (2002). The single, ancient origin of chromist plastids. Proc. Natl. Acad. Sci. U.S.A. 99, 15507–15512 10.1073/pnas.242379899 12438651 PMC137747

[B77] Yoon H. S. Müller K. M. Sheath R. G. Ott F. D. Bhattacharya D. (2006). Defining the major lineages of red algae (Rhodophyta). J. Phycol. 42, 482–492

